# Altered gut microbiota correlate with different immune responses to HAART in HIV-infected individuals

**DOI:** 10.1186/s12866-020-02074-1

**Published:** 2021-01-06

**Authors:** Yirui Xie, Jia Sun, Li Wei, Haiyin Jiang, Caiqin Hu, Jiezuan Yang, Ying Huang, Bing Ruan, Biao Zhu

**Affiliations:** 1 State Key Laboratory for Diagnosis and Treatment of Infectious Diseases, National Clinical Research Center for Infectious Diseases, Collaborative Innovation Center for Diagnosis and Treatment of Infectious Diseases, The Department of Infectious Diseases, The First Affiliated Hospital, School of Medicine, Zhejiang University, 79, QingChun Road, Hangzhou, 310003 China; 2 Ningbo Medical Center Lihuili Hospital, Ningbo, 315000 China

**Keywords:** HIV-1, Gut microbiota, Immune activation, Immunological responders, Immunological non-responders, HAART

## Abstract

**Background:**

Although gut microbiota dysbiosis has been reported in HIV infected individuals recently, the relationship between the gut microbiota and immune activation in patients with different immune responses to highly active antiretroviral therapy (HAART) is still not well understood. Gut microbiota and immune activation were studied in 36 non-HIV-infected subjects (healthy controls) and 58 HIV-infected individuals, including 28 immunological responders (IR) and 30 immunological non-responders (INR) (≥500 and < 200 CD4+ T-cell counts/μl after 2 years of HIV-1 viral suppression respectively) without comorbidities.

**Results:**

Metagenome sequencing revealed that HIV-infected immunological responders and immunological non-responders could not recover completely from the gut microbiota dysbiosis. At a 97% similarity level, the relative abundances of *Fusobacterium, Ruminococcus gnavus* and *Megamonas* were greater, whereas *Faecalibacterium, Alistipes, Bifidobacterium, Eubacterium rectale* and *Roseburia* were more depleted in the IR and INR groups than those in the healthy controls. *Ruminococcaceae* and *Alistipes* were positively correlated with nadir and current CD4+ T-cell counts, but negatively correlated with CD8 + CD57+ T-cell counts*.* Inflammation markers and translocation biomarkers (LPS) levels were positively correlated with the abundances of genera *Ruminococcus* and *Fusobacterium* but were negatively correlated with the genus *Faecalibacterium*. The relative abundances of *Escherichia-Shigella* and *Blautia* were significantly higher in the IR than those in the INR group. *Escherichia-Shigella* were negatively correlated with the CD4/CD8 ratio but positively correlated with the amount of *C*D8 + CD57+ T-cells. *Roseburia* and *Blautia* were negatively associated with nadir CD4+ T-cell and positively associated with CD8 + CD57+ T-cell counts.

**Conclusions:**

Gut microbiota dysbiosis may be one of the factors contributing to different immune responses and treatment outcomes to HAART.

**Supplementary Information:**

The online version contains supplementary material available at 10.1186/s12866-020-02074-1.

## Background

Life expectancy of individuals infected with human immunodeficiency virus (HIV) has increased enormously and HIV infection has become a chronic disease that is manageable in the combination antiretroviral therapy (ART) era. Most patients receiving ART can achieve a distinct reduction of HIV viral load and improvement of CD4+ T-cell counts compared with nadir CD4+ T-cell counts (the patient’s lowest CD4+ T-cell counts). However, the extent of immunological recovery varies greatly between individuals. The HIV-infected individuals who fail to achieve normalization of CD4+ T-cell counts despite persistent virological suppression are considered as immunological non-responders (INR) [1], which are in contrast with immunological responders (IR) [2]. As there is no unified definition of INR, the prevalence of INR ranges from 15 to 30% in the ART cohorts [3–5]. The INR were defined as patients whose absolute value of CD4+ T cells counts were less than 200 cells/μl after years of ART, although 350 cells/μl were also used as a cutoff value in the literature [6]. In contrast, IR were defined as patients whose CD4+ T-cell counts were greater than 500 cells/μl after receiving ART for years.

Emerging evidence suggests that the gut microbiome of HIV-infected patients are different from that of HIV-uninfected individuals [7]. However, these studies have not considered the immune response to ART and there have been little research focusing on patients in China [7]. Recently, several studies have reported that gut microbiota is associated with CD4+ T-cell recovery in HIV-infected patients [8–10]. The microbiome of chronic HIV infected individuals was studied in China. However, these patients had heterogeneous HIV progressions and immune responses to ART [11, 12]. Studies showed that HIV-mediated destruction of gut mucosa could lead to local and systemic inflammation [13]. Moreover, chronic inflammation was reported to be associated with the gut microbiome in the non-AIDS population [14]. These studies suggest that the gut microbiome may play a part in the immune activation in HIV-infected individuals with ART [13, 15–17]. Despite many studies of the microbiome in HIV-infected patients, there have been relatively few reports discussing the gut microbiome that occur in patients with different immune responses to ART [18, 19]. Therefore, a comparative study of the gut microbiome and immune activation was conducted on HIV patients with different immune responses to ART and the results are presented in this study. 16S ribosomal RNA (rRNA) targeted sequencing and flow cytometry were used to characterize the gut microbiome and their relationship with immune activation among immunodiscordant and immunoconcordant patients with long-term suppressive ART.

## Results

### Clinical characteristics and pyrosequencing data summary

The characteristics of all 28 IR patients, 30 INR patients and 36 healthy controls, including demographics, clinical characteristics, and pyrosequencing results are presented in Table 1. There is no significant difference between the rate of the transmission route in the IR and INR groups (*p* = 0.779). The rate of homosexual (MSM) transmission route is 57.1% vs 51.7% in the INR and IR groups, whereas the rate of the heterosexual transmission route is 20.7% vs 21.4%, and other rates are missing from their records. The viral load of all HIV-infected individuals with ART is not detected. Nadir and current CD4+ T cell counts are significantly higher in the IR group than the INR group (Table 2). No differences in the duration of ART and ongoing ART medications are observed between the IR and the INR groups. Other characteristics such as gender, age and body mass index (BMI) are generally matched among the IR, INR and healthy controls. 3,549,077 high-quality sequences in total were obtained (average sequence length 440 bp) from 94 participants. Thirty-eight thousand eight hundred forty-nine sequences per sample on average were obtained from the healthy controls, while 35,947 and 38,134 sequences per sample were obtained respectively from the IR and INR patients. Rarefaction was conducted on the OTU (Operational taxonomic unit) table to 30,174 reads per sample to avoid methodological artefacts. Specifically, 609 OTUs are defined in the healthy controls, while 486 OTUs and 567 OTUs in the IR and INR groups are defined relatively at a 97% similarity level. Significant differences of bacterial diversity (Shannon, Simpson, and Sobs), richness (ACE, Chao1) and Good’s coverage are observed among the three groups, while no significant difference are found between the IR and the INR groups. A summary is shown in Table 2.

**Table 1:** Clinical characteristics data summary

		HIV ART (+)		*P*-value
	Health Control	Immune Responders (IR)	Immune Non-responders (INR)	
Number of subjects	36	28	30	
Gender male/female	33/3	25/3	29/1	
Age (mean ± SD)	33.11 ± 3.95	36.64 ± 10.2	36.6 ± 7.19	NS
BMI (mean ± SD)	21.42 ± 3.27	21.06 ± 2.37	20.67 ± 2.74	NS
Smoking	1	0	1	NS
**Transmission, no.**				
Heterosexual	NA	6	6	NS
Homosexual transmission	NA	16	15	NS
Data Missing	NA	6	8	NS
HAART months (mean ± SD)	NA	37.25 ± 13.61	34.00 ± 10.24	NS
**Ongoing cART regimen, no. patients (%)**				
NNRTI based	NA	25	27	NS
PI based	NA	3	3	NS

*NA* (not available), *NS* (no significant) indicates *p*-value > 0.05. *NNRTI* Non-nucleoside reverse transcriptase inhibitors, *PI* Protease inhibitor

**Table 2:** Cellular immune activation markers sequencing data summary

T cell markers		HIV ART (+)		*P*-value
	Health Control	Immune Responders (IR)	Immune Non-responders (INR)	
Nadir CD4^+^ T cells (mean ± SD)	NA	309.89 ± 128.81	95.23 ± 108.92	< 0.0001
Current CD4 ^+^ T cells (mean ± SD)	NA	608.30 ± 158.25	230.5 ± 87.50	< 0.0001
Current CD4+/CD8+ T-cell ratio	NA	0.8 ± 0.36	0.35 ± 0.20	< 0.0001
HIV RNA	NA	ND	ND	
%CD4 + HLADR+CD38+	NA	7.72 ± 4.30	10.30 ± 10.45	NS
%CD4 + CD25+	NA	1.26 ± 0.99	1.09 ± 1.04	NS
%CD4 + CD57+	NA	2.57 ± 1.92	2.43 ± 3.30	NS
%CD8 + HLADR+CD38+	NA	20.68 ± 11.35	23 ± 12.39	NS
%CD8 + CD57+	NA	14.36 ± 7.11	23.98 ± 12.30	0.001
**Cytokines**				
IL-2(pg/mL, mean ± SD)	9.47 ± 6.79	120.96 ± 113.96	153.81 ± 118.79	< 0.0001
IL-4(pg/mL, mean ± SD)	13.49 ± 31.01	34.52 ± 43.30	32.51 ± 31.17	0.009
IL-6(pg/mL, mean ± SD)	8.39 ± 12.57	126.90 ± 95.33	130.82 ± 60.62	< 0.0001
IL-9(pg/mL, mean ± SD)	3.68 ± 6.20	94.00 ± 78.07	96.57 ± 48.71	< 0.0001
IL10(pg/mL, mean ± SD)	2.48 ± 2.35	44.69 ± 37.66	51.32 ± 31.65	< 0.0001
IL-13(pg/mL, mean ± SD)	85.45 ± 65.02	96.76 ± 71.54	82.94 ± 62.96	NS
IL-17A (pg/mL, mean ± SD)	26.22 ± 52.89	133.41 ± 161.61	94.80 ± 83.84	< 0.0001
IL-17F (pg/mL, mean ± SD)	5.45 ± 5.68	19.15 ± 19.48	20.59 ± 20.34	< 0.0001
IL-21(pg/mL, mean ± SD)	41.33 ± 48.87	104.43 ± 88.11	93.84 ± 56.89	0.002
IL-22(pg/mL, mean ± SD)	153.98 ± 97.15	291.60 ± 176.36	293.10 ± 125.37	0.001
IFN-γ (pg/mL, mean ± SD)	12.55 ± 29.52	81.57 ± 84.50	69.36 ± 83.42	< 0.0001
TNF-α (pg/mL, mean ± SD)	8.39 ± 15.57	38.86 ± 44.19	32.22 ± 26.47	< 0.0001
LPS (pg/mL, mean ± SD)	24.22 ± 18.88	76.55 ± 40.05	104.98 ± 56.15	< 0.0001^b^
sCD14(pg/mL, mean ± SD)	1583.60 ± 292.80	2480.42 ± 999.88	2142.65 ± 496.53	< 0.0001
**Pyrosequencing data**				
Sobs index^a^	194.11 ± 47.45	116.89 ± 39.71	118.11 ± 46.96	< 0.0001
Shannon index^a^	3.14 ± 0.53	2.47 ± 0.54	2.44 ± 0.56	< 0.0001
Simpson index^a^	0.12 ± 0.09	0.19 ± 0.11	0.19 ± 0.13	< 0.0001
ACE^a^	223.97 ± 53.59	147.26 ± 39.98	148.41 ± 49.99	< 0.0001
Chao 1 index^a^	224.29 ± 54.76	144.52 ± 45.25	145.37 ± 51.01	< 0.0001
Good’s coverage (%) ^a^	99.82 ± 0.04	99.87 ± 0.04	99.87 ± 0.04	< 0.0001

^a^Indicates that the alpha diversity was calculated after the reads number of each sample were equalized. *NA* (not available), *ND* (not detected), *NS* (no significant) indicates *p*-value > 0.05, ^b^compared between the IR and the INR group

### Compositional analysis of fecal microbiota

**Fig. 1:**
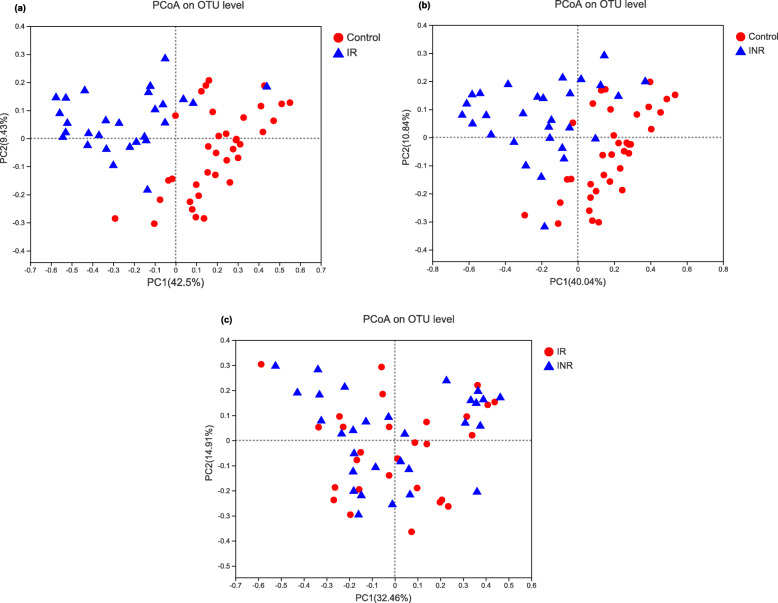
Principal coordinates analysis (PCoA) of microbiomes in the patients and the healthy controls. Remarkable differences of bacterial communities among the immunological responders (IR), immunological non-responders (INR) and the healthy controls (**a**, **b**); no significant difference between the IR and INR groups (**c**)

Principal coordinate analysis (PCoA) by weighted UniFrac matrices shows obvious differentiation of bacterial communities between the IR and the healthy controls (PERMANOVA, pseudo-F: 8.99, *R*^*2*^ = 0.13, *P* = 0.001, Fig. 1a), the INR and the healthy controls (PERMANOVA, pseudo-F: 8.77, *R*^*2*^ = 0.12, *P* = 0.001, Fig. 1b), while no significant differences are observed between the IR and INR groups (PERMANOVA, pseudo-F: 0.80, *R*^*2*^ = 0.01, *P* = 0.71, Fig. 1c).

**Fig. 2:**
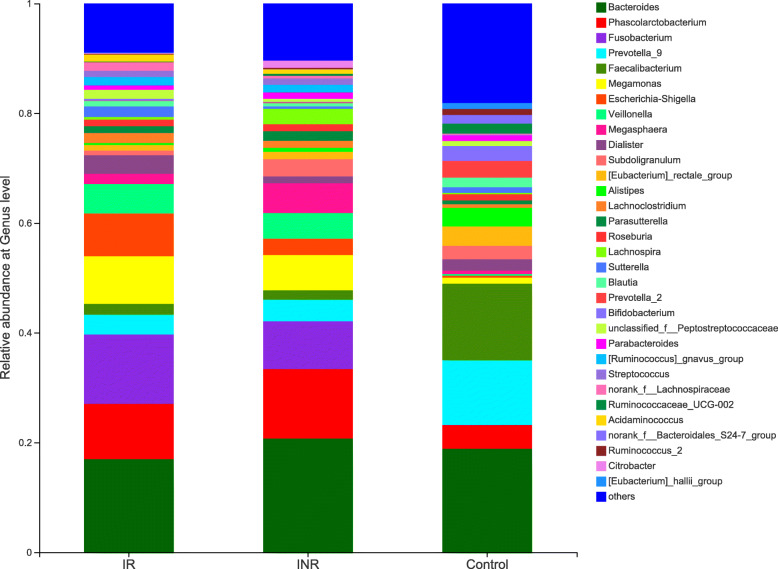
The relative abundance of fecal microbiota at the genus level in the patients and the healthy controls. IR: immunological responders; INR: immunological non-responders; Control: healthy controls

The data of average relative abundances of each bacterial phyla and genera in patients and the healthy controls are showed respectively in the figures (Fig. 2; S1, S2 and S3 Fig) and Table (S1 Table). The Wilcoxon rank sum test was used to detect taxa with significant differences in relative abundances among groups (confidence interval method). At the phylum level, Bacteroidetes, Actinobacteria, Tenericutes and Lentisphaerae are more abundant in the healthy controls than in the IR group. The relative abundances of Proteobacteria, Fusobacteria and Saccharibacteria are significantly higher in the IR group (S1 Fig. 2a) than those in the healthy controls. The relative abundances of 11 families are significantly different between the IR and the healthy controls. The relative abundances of 93 genera, including 15 predominant (> 1% of the total sequences in either group) and 78 less-predominant genera, are significantly different between the healthy controls and the IR groups. Among the different predominant genera, *Lachnoclostridium*, *Megasphaera*, *Escherichia-Shigella*, *Veillonella*, *Streptococcus*, *Fusobacterium*, and *Ruminococcus gnavus* are found to be overrepresented in the IR group. The relative abundances of *Faecalibacterium, Eubacterium rectale, Alistipes, Subdoligranulum, Bifidobacterium, Roseburia, Ruminococcaceae* and *Parasutterella* are higher in the healthy controls (Fig. 2 and S1 Fig. 2b) than in the IR group. A taxonomy-based bacterial comparison was conducted to define the differences between the healthy controls and the INR groups. At the phylum level, Bacteroidetes, Actinobacteria, Lentisphaerae are more abundant in the healthy controls than those in the INR group, while Proteobacteria, Fusobacteria, Tenericutes, Saccharibacteria and unclassified k norank are more abundant in the INR group than those in the healthy controls (Fig. 2 and S1 Fig. 2c). At the genus level, the relative abundances of 83 genera (including 11 predominant genera) are different between the healthy controls and the INR groups. The relative proportions of *Faecalibacterium, Eubacterium rectale, Alistipes, Bifidobacterium*, *Blautia, Roseburia* and *Ruminococcaceae* are more abundant in the healthy controls than those in the INR group. *Parasutterella, Megasphaera, Fusobacterium, and Ruminococcus gnavus* are found to be overrepresented in the INR group (Fig. 2 and S1 Fig. 2d). Although there is no significant difference between the IR and the INR group at the phylum level (S1 Fig. 2e), the abundances of 12 genera (including 2 predominant genera) are different between the IR and INR groups. The abundances of the two predominant genera *Escherichia-Shigella* and *Blautia* are significantly higher in the IR than those in the INR group (Fig. 2 and S1 Fig. 2f).

In order to identify the key phylotypes responsible for the difference found in distinguishing the fecal microbiota of different groups, linear discriminant analysis (LDA) effect size (LEfSe) was performed and a threshold of three on effect size was used. Taxonomic cladograms which represents the microbiota structure and predominant bacteria in the three groups is presented, and the biggest differences between the two communities are presented in S4 Fig.

### Comparison of the T-cell activation in the IR and INR groups

As expected, nadir CD4+ T-cell, current CD4+ T-cell counts and CD4/CD8 ratio are lower in the INR group than those in the IR group (*p* < 0.0001). The proportion of CD8 + CD57+ T-cell in the INR group is significantly lower than those in the IR group (*p* < 0.001). The proportion level of CD4+ and CD8+ T-cells immune activation (CD4/8+ T-cell by the expression of CD25+, HLA-DR+, and HLA-DR+/CD38+) is similar in the INR and IR groups (Table 1).

### Comparison of the bacterial translocation markers and inflammation profiles

Lipopolysaccharide (LPS), which translocates from the gut to the blood stream, is commonly used as the major antigens to drive the chronic immune activation. The level of LPS is significantly higher in the INR group compared with other groups (*p* < 0.0001). However, the soluble immune activation marker sCD14 shows no difference between the groups. Of the 13 markers studied, the level of IL-13 shows no difference among groups while the other 12 markers (IL-2, IL-4, IL-5, IL-6, IL-9, IL10, IL-17A, IL-17F, IL-21, IL-22, IFN-γ and TNF-α) appear to be significantly higher in the INR and IR groups when compared with the healthy controls, but there is no significant difference between the INR and IR groups (Table 1).

### Association between fecal microbiota and immune activation

**Fig. 3:**
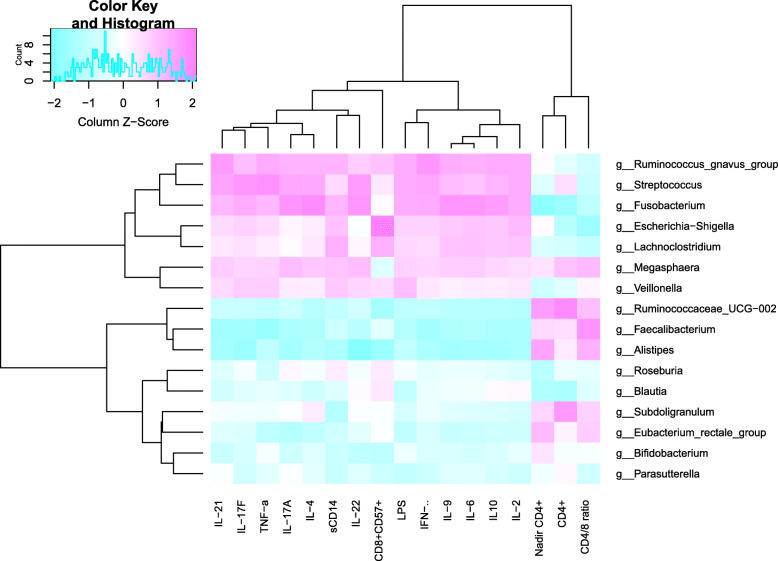
The composition of fecal microbiota correlates with the markers of immune activation. Some cellular and soluble markers of immune activation was in correlation with specific genera of gut microbiota. Spearman’s correlation was used. Associations with theBenjamini – Hochberg adjusted *p*-value lower than 0.01 were considered relevant

Spearman correlations of the relative abundances of bacteria genera and levels of T-cell activation, inflammation or translocation biomarkers are evaluated (Fig. 3). Interestingly, nadir CD4+ T-cell counts are positively correlated with the abundances of *Ruminococcaceae* and *Alistipes*, while current CD4+ T-cell counts are strongly positively correlated with the abundances of *Ruminococcaceae* and *Subdoligranulum*. The genus *Fusobacterium* is negatively correlated with nadir and current CD4+ T-cell. The CD4/CD8 ratio is positively correlated with the genera *Faecalibacterium* and *Ruminococcaceae,* but negatively correlates with *Escherichia-Shigella*. Moreover, the CD8 + CD57+ T-cell counts is positively correlated with *Escherichia-Shigella* but negatively correlates with the genera *Ruminococcaceae* and *Alistipes*. The genera *Roseburia* and *Blautia* are negatively associated with nadir CD4+ T-cell and positively associated with CD8 + CD57+ T-cell counts. Inflammation markers and LPS are positively correlated with the *Ruminococcus* and *Fusobacterium* but negatively correlates with the genus *Faecalibacterium* (Fig. 3).

## Discussion

The objective of highly active antiretroviral therapy (HAART) is to achieve immune reconstitution and successful viral suppression in HIV-infected patients [2]. Even with complete viral suppression, the CD4+ T cell cannot be reversed completely and microbial translocation continues after peripheral CD4+ T cell restoration [20–23]. Gut microbiota has been reported to have critical impact on human biology and pathophysiology. The gut microbiota’s function as a key factor in the process of immune homeostasis is accepted widely by the research community now [23]. Compositional and functional changes of the gut microbiota have indicated a new relationship between the gut bacterial community and immunity in HIV-infected individuals with treatments [7, 24–26]. However, the way the microbiome contributes to immune response in HIV positive individuals is poorly understood. So, the objective of this study is to investigate the relationship between the fecal microbiome and immune activation in HIV-positive individuals with different immune responses to long-term ART in China.

The microbiome dysbiosis in HIV-infected individuals has been introduced in several studies [7, 25, 26]. However, early studies only adopted a small amount of IR and INR patients as subjects [8, 10] and did not taken immune response to HAART into consideration [27]. The previous studies showed that the relative abundance of *Fusobacterium* was independently associated with poorer CD4+ T-cell recovery [10], and the relative abundances of unclassified *Subdoligranulum sp*. and *C. comes* were positively correlated with CD8 + HLA-DR+ T-cell counts and CD8 + HLADR+/CD8+ percentage in the HIV-infected subjects [8]. The current study has adopted higher number of IR and INR patients and have expanded the range of observations when investigating the relationship between the gut microbiota and immune activation. This study found that the ecological indices of microbiota (including community diversity, richness and observed species numbers) were significantly lower in the IR and INR groups when compared with the healthy controls. Additionally, PCoA analysis showed remarkable differentiation of bacterial communities between the IR, INR and the healthy controls. The findings suggested that HIV-infected immunological responders and immunological non-responders could not recover completely from the gut microbiota dysbiosis.

Furthermore, this study found that the IR and INR group had a unique bacterial signature at the phylum level. Proteobacteria, Fusobacteria, and Saccharibacteria were more abundant, whereas Bacteroidetes, Actinobacteria and Lentisphaerae were depleted in the IR and INR groups. At the 97% similarity level, the predominant genera *Fusobacterium, Ruminococcus gnavus* and *Megamonas* were more abundant, whereas *Faecalibacterium, Alistipes, Bifidobacterium, Eubacterium rectale*, and *Roseburia* were depleted in the IR and INR groups compared to the healthy controls. Different taxa from bacterial phyla Proteobacteria, Fusobacteria and Bacteroidetes between HIV positive and negative individuals have been reported in related studies. The result of this study indicates that the Proteobacteria were more abundant in HIV-infected individuals, which is consistent with most of the published studies, while other studies report no change [8, 10–12, 28–33]. The family Enterobacteriaceae was reported positively associated with markers of monocyte activation (sCD14), inflammation and colonic T cell activation and inversely correlated with blood CD4+ T-cell counts [30, 32, 34]. *Escherichia-Shigella,* which belonged to Enterobacteriaceae, were negatively correlated with the CD4/CD8 ratio, but positively correlated with the CD8 + CD57+ T-cell which was the hallmark of immunosenescence in HIV infection [35, 36]. Furthermore, *Escherichia-Shigella* was more abundant in the IR group than that in INR group in this study.

The phylum Fusobacteria (mostly driven by its constituent genus *Fusobacterium*) was reported to be associated with intestinal inflammation [37, 38] and was abundant in HIV-infected individuals in some studies [29, 33, 39, 40]. Here, this study found increased abundance of *Fusobacterium* in the IR and INR groups. Furthermore, inflammation markers and translocation biomarkers (LPS) were positively correlated with *Fusobacterium*, while the nadir and current CD4+ T-cell counts were negatively correlated with *Fusobacterium* in this study. This was consistent with the previous published studies which reported that the relative abundance of *Fusobacterium* was independently associated with poorer CD4 T-cell recovery and the enrichment of *Fusobacterium* was associated with decreased ability of immune recovery and persistent immune dysfunction following ART [10].

The phylum Bacteroidetes, which include the families Bacteroidaceae, Prevotellaceae, Porphyromonadaceae and Rikenellaceae, exhibited a more heterogeneous pattern of changes in the HIV-infected individuals [11, 12, 23, 28–34, 39–42]. The family Bacteroidaceae (mostly driven by the abundance of the genus *Bacteroides*) which was generally considered to be playing an anti-inflammatory role [43–46], was depleted in the IR and INR groups. The bile-tolerant family Rikenellaceae (mostly driven by the abundance of the genus *Alistipes*) which had protective properties against *C. difficile* infection [46, 47], was overall depleted in the IR and INR groups. Actinobacteria, which was reported to be similar in the proximal gut of HIV-infected patients and negative controls [32], were depleted in the IR and INR groups in this study. The *Alistipes* was negatively correlated with the CD8 + CD57+ T-cell and positively correlated with the nadir CD4+ T-cell counts. Notably, the commonly used probiotics *Bifidobacterium* which belong to the phylum of Actinobacteria was depleted in the IR and INR groups compared with the healthy controls [48].

Although the abundance of phylum Firmicutes was similar in all three groups, genera such as *Faecalibacterium* and *Ruminococcaceae* were depleted in the IR and INR groups compared with the healthy controls. *Ruminococcaceae* has been playing both protective and disruptive roles within the gut microbial community, such as the production of anti-inflammatory short-chain fatty acids (SCFA) [49] or the degradation of host mucus and potential proinflammatory in IBD [50]. In this study, *Faecalibacterium* and *Ruminococcaceae UCG-002*, which belong to the family of *Ruminococcaceae* were depleted in the IR and INR groups compared with the healthy controls. Interestingly, *Ruminococcaceae* was positively correlated with the current nadir CD4+ T-cell counts while negatively correlated with the CD8 + CD57+ T-cell counts. In addition, inflammation markers and LPS were positively correlated with the abundance of genus *Ruminococcus*. *Faecalibacterium* has been reported as the anti-inflammatory commensal genus [29, 51]. In this study, the genus *Faecalibacterium* was positively correlated with CD4/CD8 ratio while negatively correlated with the inflammation markers and LPS.

The normal (gut microbiota) groups which commonly present in large amounts in the healthy controls bear more significance and interest us more compared to various pathogens. Both *Roseburia* and *Blautia* (which belong to the phylum Firmicutes, class Clostridia, family Lachnospiraceae) were members of the groups of commensals like which described above and have been described as the SCFA producers with beneficial effect on the intestinal barrier and an important energy source for epithelial cells [52]. In this study, *Blautia, and Roseburia* were more abundant in the healthy controls and the abundance of *Blautia* increased significantly in the IR group than the INR group. *Roseburia* and *Blautia* were negatively associated with the nadir CD4+ T-cell and positively associated with the CD8 + CD57+ T-cell counts. These suggested that *Blautia* and *Roseburia* might be associated with the treatment outcome.

Altogether, these results indicate that immune activation in the HIV-infected patients was associated with the gut microbiota dysbiosis observed. Based upon these findings, this study speculate that the gut microbiota may be one of the factors contributing to different immune responses to HAART. *Fusobacterium, Alistipes, Ruminococcaceae, Faecalibacterium, Escherichia-Shigella, Roseburia* and *Blautia* maybe the major genera contributing to different immune responses and treatment outcome in immunodiscordant and immunoconcordant patients with long-term suppressive ART. However, we did not collect samples before the ART treatment, so future research on whether the gut microbiome composition influences treatment effect or not is still needed and this study cannot directly address the question if changes in the microbiota were causative or rather a result of systemic HIV-1-associated immune activation. This study acknowledges that the extensive dietary data and various living conditions of the subjects involved may lead to biases during the analysis. On the other hand, while correlation analysis was helpful in linking biological clues to the impact of dysbiosis in immune responses in the patients, a direct manipulation of the microbiome was needed to validate their cellular and biochemical actions in vitro or in vivo, and the exact mechanism of how HIV infection can lead to dysbiosis in the gut need to be studied in the future.

## Conclusions

In summary, this study presents the research results regarding the gut microbiota dysbiosis in HIV-infected immunological non-responders and immunological responders, and concludes that the gut microbiota dysbiosis may be one of the factors contributing to different immune responses to HAART. *Fusobacterium, Alistipes, Ruminococcaceae, Faecalibacterium*, *Escherichia-Shigella, Roseburia and Blautia* may be the major genera contributing to different immune responses and treatment outcome in immunodiscordant and immunoconcordant patients with long-term suppressive ART.

## Methods

### Recruitment of subjects

The process of participants’ recruitment and sample collection is stated in Suppl. S5 Fig. 5. 8 HIV-infected individuals in total, including 28 immunological responders (IR), 30 immunological non-responders (INR), and 36 healthy subjects (healthy controls) were recruited. IR and INR were defined as patients whose CD4+ T-cell counts/μl equal or is more than 500 or less than 200 after 2 years of receiving complete viral suppression therapy respectively. Subjects were recruited from the HIV clinic of the First Affiliated Hospital of Zhejiang University from November 2015 to October 2017. All HIV-positive participants were diagnosed by the Disease Control and Prevention Center of Zhejiang Province. All HIV-positive subjects were on two nucleoside reverse transcriptase inhibitors (NRTIs) + nonnucleoside reverse transcriptase inhibitors (NNRTIs) or the protease inhibitor-based therapy: Zidovudine/Tenofovir Disoproxil Fumarate (AZT/TDF) + Lamivudine (3TC) + Efavirenz (EFV) or Lopinavir/ritonavir (LPV/r). The healthy controls were all healthy HIV-negative volunteers and most of them belong to the staff of the institution. Age, gender, and body mass index (BMI) of the healthy controls are similar to those of the HIV-positive individuals (Table 1). Candidates with following conditions and traits are excluded from the subject selection: age over 60 years old; having opportunistic infection; having hepatitis B or C infection; having history of using antibiotics, immunosuppressive regimen, probiotics, prebiotics, or symbiotics in the previous 6 months; having used rectally administered medications within 48 h before selection; BMI > 30; having a history of inflammatory bowel disease (IBD); having active inflammation affecting the gastro intestines.

### Ethics statement

All participants provided written informed consents before participating in the study. This study conforms to the ethical norms of the 1975 Helsinki Declaration. The research protocol was approved by the Institutional Review Committee of the First Affiliated Hospital of Zhejiang University on October 7, 2015. All the data used for analysis were anonymized.

### Fecal samples collection and DNA extraction

Fecal samples of the participants were collected in sterile container before their clinic visits and were stored in a − 80 °C environment before the DNA extraction. The DNA was extracted using a QIAamp DNA stool mini kit (QIAGEN, Hilden, Germany) and glass-bead beat on Mini-bead beater (FastPrep; Thermo Electron Corporation, Boston, MA, USA), following the manufacturer’s instructions. The DNA’s quantification and purity were assessed by NanoDrop ND-1000 spectrophotometer (Thermo Electron Corporation). The integrity and sizes of the DNA were reviewed by 1.0% agarose gel electrophoresis. The DNA was stored at a − 20 °C environment for further analysis.

### Polymerase chain reaction (PCR) and 16S rRNA gene sequencing

PCR amplification of the bacterial 16S rRNA gene V3–V4 region was performed using universal primers (338F 5′- ACTCCTACGGGAGGCAGCAG-3′, 806R5′-GGACTACHVGGGTWTCTAAT-3′). The PCR reactions were conducted using the following program: 3 mins of denaturation at 95 °C, 27 cycles of 30s at 95 °C, 30s for annealing at 55 °C, and 45 s for elongation at 72 °C, and a final extension at 72 °C for 10 mins. PCR reactions were performed in a triplicate 20 μl mixture containing 4 μl of 5 × FastPfu Buffer, 2 μl of 2.5 mM dNTPs, 0.8 μl of each primer (5 μM), 0.4 μl of FastPfu Polymerase and 10 ng of the template DNA. The resulted PCR products were extracted from a 2% agarose gel and then purified using the AxyPrep DNA Gel Extraction Kit (Axygen Biosciences, Union City, CA, USA) and quantified using QuantiFluor™-ST (Promega, USA) following the manufacturer’s protocol. Purified amplicons were pooled in equimolar and paired-end sequenced (2 × 300) on an Illumina MiSeq platform (Illumina, San Diego, USA) following the standard protocols by Majorbio Bio-Pharm Technology Co. Ltd. (Shanghai, China). The raw reads were deposit into the NCBI Sequence Read Archive (SRA) database and is accessible with the following link: https://www.ncbi.nlm.nih.gov/sra/PRJNA533202.

### Bioinformatics and statistics

The raw fastq files were demultiplexed and quality-filtered using Trimmomatic, and then merged by FLASH. The original reads were trimmed with a minimum length of 50 bp with an average quality score of 20. Two mismatches were allowed in primer sequences and reads containing ambiguous bases were removed. Ten homopolymers authorized in sequences were limited. OTUs were picked at a 97% similar threshold by UPARSE (http://drive5.com/uparse/) and chimera identification sequence was performed using UCHIME. Taxonomy-based analyses were performed by RDP Classifier algorithm (http://rdp.cme.msu.edu/) in combination with 16 s rRNA Silva128 database with 70% cut-off confidence.

### Viral load, flow Cytometry and Immunophenotype

Quantifications of CD4+ and CD8+ T-cells as well as HIV-1 RNA were carried out in HIV-infected individuals using flow cytometry and Cobas Amplicor (Roche Molecular Systems Inc., Branchburg, New Jersey, USA) as the clinical routine. The percentage of CD4+ and CD8+ T cells expressing markers of activation (CD25+, CD38+, HLADR+, or CD38+/HLA-DR+) and senescence (CD57+) were quantified by the BD FACS Canto II flow cytometer (BD Biosciences, California, USA) using fresh anticoagulated whole blood. Antibody such as CD3-FITC, CD4- PerCP/Cy5.5, CD8-Brilliant Violet 510™, CD38-Brilliant Violet 421, CD25-PE, HLA-DR-APC/Fire™ 750, and CD57-allophycocyanin (APC) were purchased from Biolegend (San Diego, CA).

### Bacterial translocation and immune activation markers

Sera samples of 27 IR, 30 INR, and 17 healthy participants were collected for the measurement of the immune activation markers. These markers were quantified using LEGENDplex™ Human Th Cytokine Panel (Biolegend, San Diego, CA): IL-2, IL-4, IL-6, IL-9, IL10, IL-13, IL-17A, IL-17F, IL-21, IL-22, TNF-α and interferon (IFN)-γ, in line with the manufacturer’s instructions. Human Lipopolysaccharides (LPS) ELISA Kit (CUSABIO; Wuhan, China) and Human soluble CD14 (sCD14) ELISA Kit (MultiSciences, Hangzhou, China) were used to test the plasma LPS and sCD14 following the standard protocols. Two replicates were performed for each assay.

### Statistics analysis

OTUs that reached the 97% level of nucleotide similarity level were used for alpha diversity (Shannon, Simpson, and Sobs), richness (ACE and Chao1), Good’s coverage, and rarefaction curve, and phylogenetic beta diversity measures analyses by mothur. PERMANOVA (permutational multivariate analysis of variance) were used to assess beta diversity based on the UniFrac distances. The number of permutations was 999 for PERMANOVA. The results were imported into Phyloseq for subsampling normalization, manipulation, and graph visualization by R (V.3.1.3, The R Project for Statistical Computing) [53]. Linear discriminant analysis (LDA) effect size (LEfSe) used the Kruskal-Wallis rank sum test to detect features with significant different abundances between the assigned taxa. LEfSe is available online in the Galaxy workflow framework [54]. Principal Coordinates analysis (PCoA) was conducted for weighted Unifrac data to visualize the microbial communities. The one-way ANOVA, non-parametric test, Wilcoxon rank sum test and Mann-Whitney U test which used for comparisons between groups were conducted in the R package and SPSS software (version 21, SPSS, Inc., Chicago, IL, USA). The average abundance values for each bacterium are depicted as mean ± SD. A significant alpha of 0.05 and LDA effect size threshold of 3 were used for all biomarkers. Correlations between the variables were calculated using the Spearman’s rank-correlation analysis by R package; and associations with the Benjamini-Hochberg adjusted *p*-value lower than 0.01 were considered relevant.

## Supplementary Information

**Additional file 1: Figure S1.** Taxonomic differences of fecal microbiota between the patients and healthy controls groups. Comparison of relative abundances at the bacterial phylum (a, c, e) and genus (b, d, f) levels between the immunological responders (IR), immunological non-responders (INR) and the healthy controls (Control) group. # indicates *P* < 0.05. * indicates *P* < 0.01. The average abundance values for each bacterium are depicted as mean ± SD.

**Additional file 2: Figure S2.** The relative abundance bar chart figure of each sample at the phylum level. IR: immunological responders; INR: immunological non-responders; Control: healthy controls.

**Additional file 3: Figure S3.** The relative abundance bar chart figure of each sample at the genus level. IR: immunological responders; INR: immunological non-responders; Control: healthy controls.

**Additional file 4: Figure S4.** Taxonomic differences of fecal microbiota between the immunological responders (IR), immunological non-responders (INR) and healthy controls (Control) group. Cladogram representing the features that are discriminative using the LDA model results on the bacterial hierarchy (a, c, e). LDA coupled with effect size measurements identifies the most differentially abundant taxon between the two groups (b, d, f).

**Additional file 5: Figure S5.** The recruitment of participants and the process of sample collection.

**Additional file 6: Table S1.** The fecal microbiota relative abundance of each sample in different groups at the genus level.

## Data Availability

The raw reads were deposited into the NCBI Sequence Read Archive (SRA) database and is accessible with the following link: https://www.ncbi.nlm.nih.gov/sra/PRJNA533202.

## Abbreviations

HIV: Human immunodeficiency virus
ART: Antiretroviral therapy
HAART: Highly active antiretroviral therapy
INR: Immunological non-responders
IR: Immunological responders
BMI: Body mass index
MSM: Men who have sex with men
SCFA: Short-chain fatty acids
LPS: Lipopolysaccharide
PCoA: Principal coordinate analysis
LDA: Linear discriminant analysis
LEfSe: Linear discriminant analysis effect size
OTU: Operational taxonomic unit
16S rRNA: 16S ribosomal RNA
NRTIs: Nucleoside reverse transcriptase inhibitors
NNRTIs: Nonnucleoside reverse transcriptase inhibitors
AZT: Zidovudine
TDF: Tenofovir Disoproxil Fumarate
3TC: Lamivudine
EFV: Efavirenz
LPV/r: Lopinavir/ritonavir
