# Supplementary figures and images for: Altered gut microbiota correlate with different immune responses to HAART in HIV-infected individuals
Source: BMC Microbiol. 2021 Jan 6;21:11. doi: 10.1186/s12866-020-02074-1 (PMC7789785; doi:10.1186/s12866-020-02074-1)

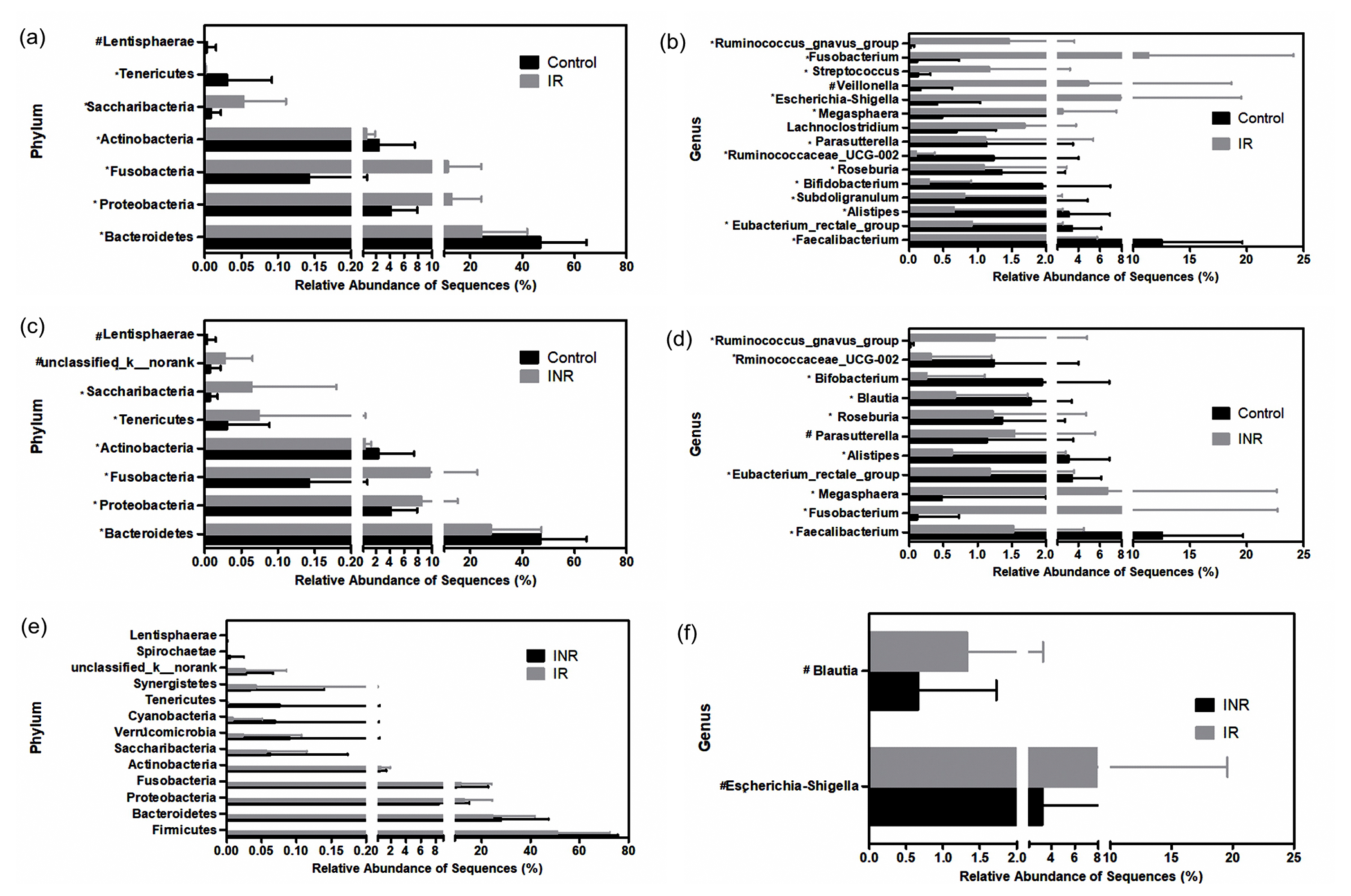

Supplement: Supplementary file 1 — Additional file 1: Figure S1. Taxonomic differences of fecal microbiota between the patients and healthy controls groups. Comparison of relative abundances at the bacterial phylum (a, c, e) and genus (b, d, f) levels between the immunological responders (IR), immunological non-responders (INR) and the healthy controls (Control) group. # indicates P < 0.05. * indicates P < 0.01. The average abundance values for each bacterium are depicted as mean ± SD. [file 12866_2020_2074_MOESM1_ESM.png]

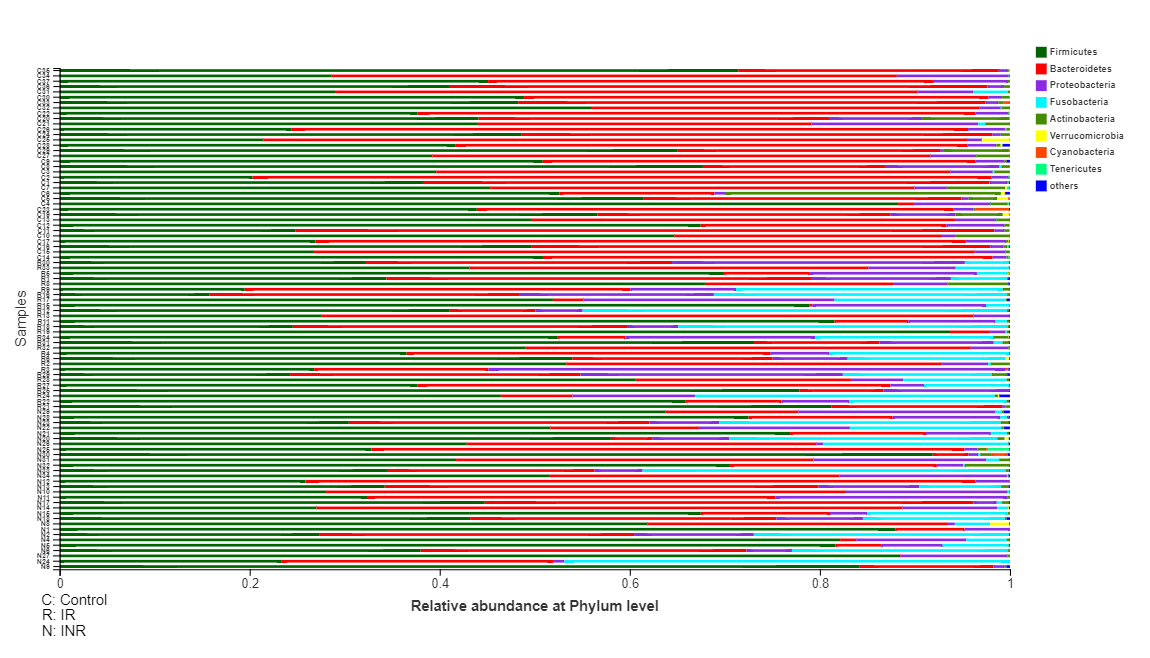

Supplement: Supplementary file 2 — Additional file 2: Figure S2. The relative abundance bar chart figure of each sample at the phylum level. IR: immunological responders; INR: immunological non-responders; Control: healthy controls. [file 12866_2020_2074_MOESM2_ESM.png]

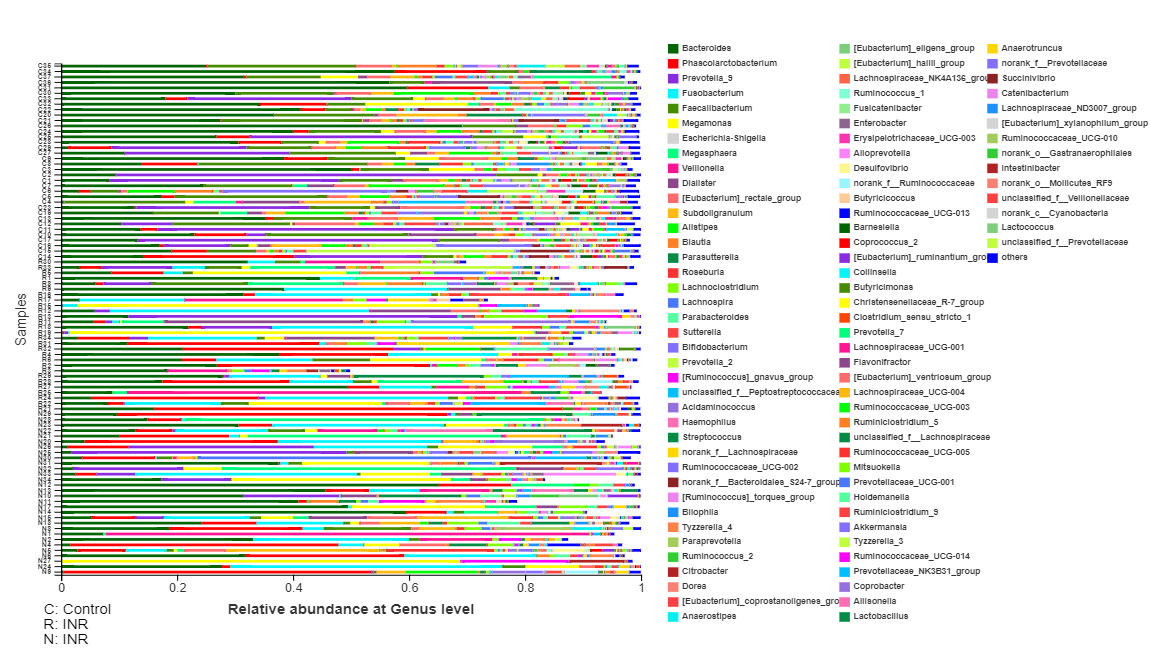

Supplement: Supplementary file 3 — Additional file 3: Figure S3. The relative abundance bar chart figure of each sample at the genus level. IR: immunological responders; INR: immunological non-responders; Control: healthy controls. [file 12866_2020_2074_MOESM3_ESM.png]

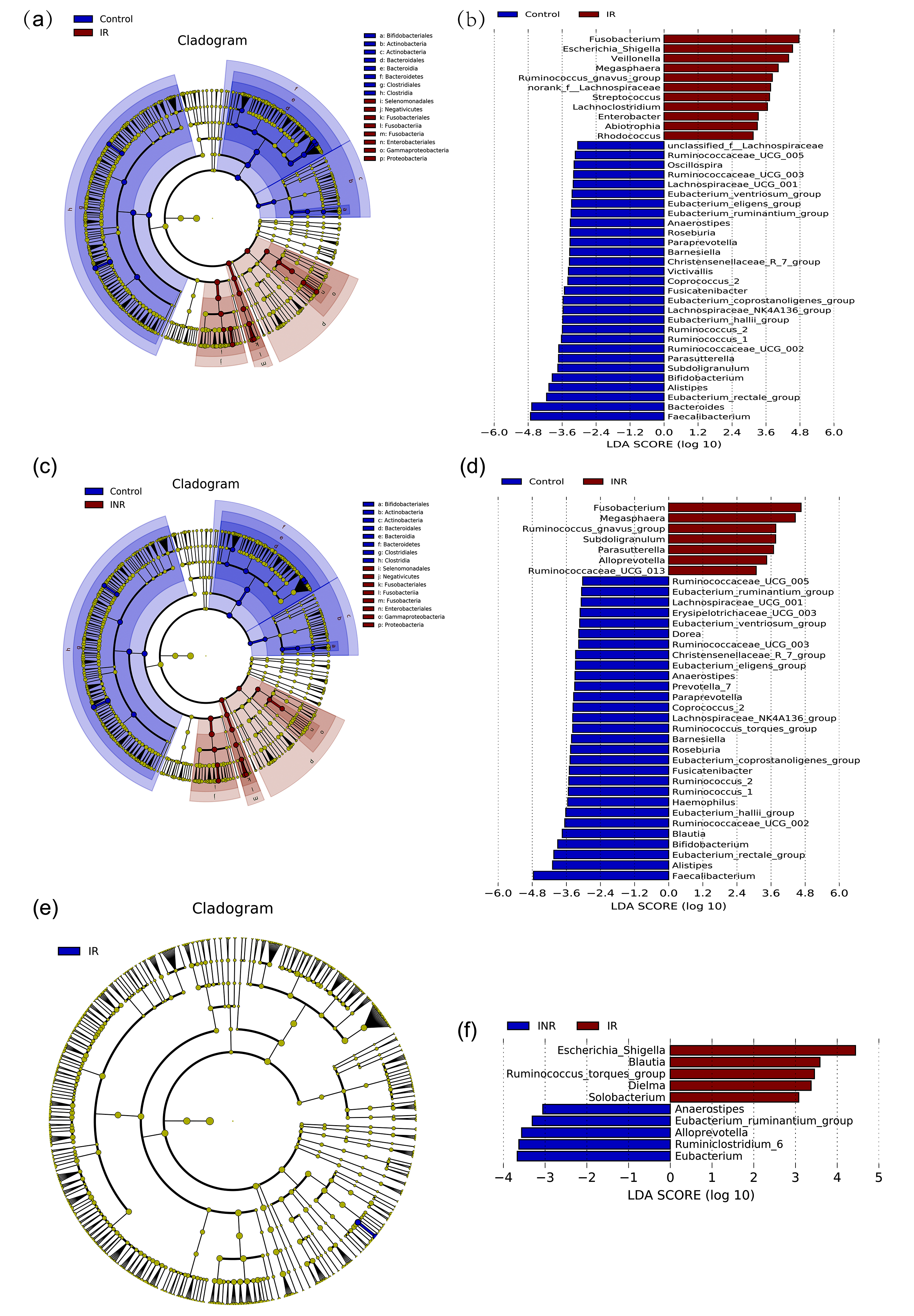

Supplement: Supplementary file 4 — Additional file 4: Figure S4. Taxonomic differences of fecal microbiota between the immunological responders (IR), immunological non-responders (INR) and healthy controls (Control) group. Cladogram representing the features that are discriminative using the LDA model results on the bacterial hierarchy (a, c, e). LDA coupled with effect size measurements identifies the most differentially abundant taxon between the two groups (b, d, f). [file 12866_2020_2074_MOESM4_ESM.png]

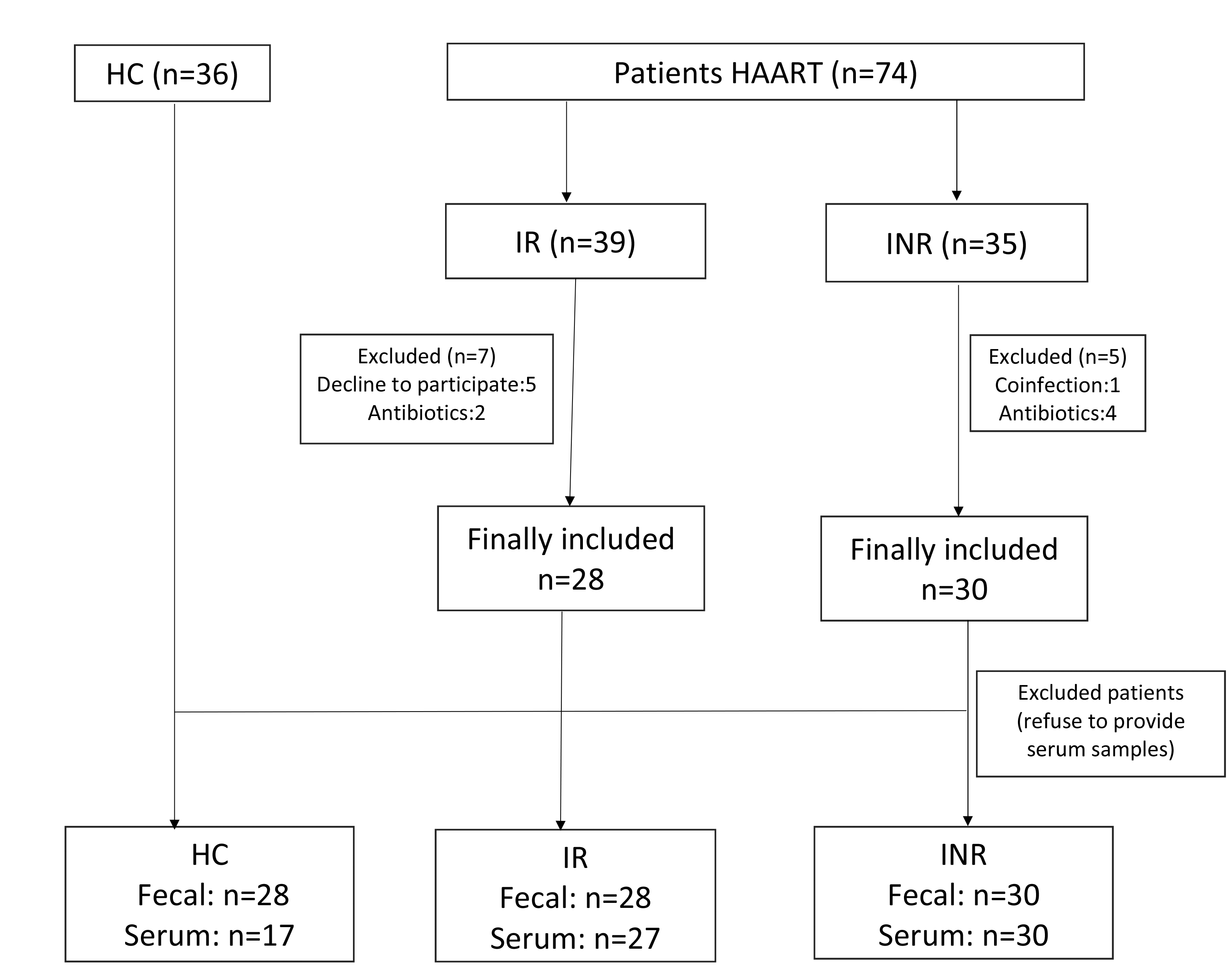

Supplement: Supplementary file 5 — Additional file 5: Figure S5. The recruitment of participants and the process of sample collection. [file 12866_2020_2074_MOESM5_ESM.png]
